# The addition of vildagliptin to metformin prevents the elevation of interleukin 1ß in patients with type 2 diabetes and coronary artery disease: a prospective, randomized, open-label study

**DOI:** 10.1186/s12933-017-0551-5

**Published:** 2017-05-22

**Authors:** Arwa Younis, Dana Eskenazi, Ronen Goldkorn, Jonathan Leor, Nili Naftali-Shani, Enrique Z. Fisman, Alexander Tenenbaum, Ilan Goldenberg, Robert Klempfner

**Affiliations:** 1 The Leviev Heart Center, Sheba Medical Center, Tel Hashomer, Sheba Road 2, 52620 Ramat Gan, Israel; 2 Sackler School of Medicine, Tel Aviv University, Tel Aviv, Israel; 3 Cardiovascular Diabetology Research Foundation, Holon, Israel; 4 Heart Research Follow-up Program, University of Rochester, Rochester, NY USA

**Keywords:** Interleukin 1 beta, Vildagliptin, Dipeptidyl peptidase-4 inhibitors, Gliptins, Metformin

## Abstract

**Background:**

Patients with type 2 diabetes present with an accelerated atherosclerotic process. Animal evidence indicates that dipeptidyl peptidase-4 inhibitors (gliptins) have anti-inflammatory and anti-atherosclerotic effects, yet clinical data are scarcely available.

**Design and methods:**

A prospective, randomized, open-label study was performed in 60 patients with coronary artery disease (CAD) and type 2 diabetes, who participated in a cardiac rehabilitation program. After a washout period of 3 weeks, patients were randomized in a 2:1 ratio to receive combined vildagliptin/metformin therapy (intervention group: n = 40) vs. metformin alone (control group: n = 20) for a total of 12 weeks. Blinded assessment of interleukin-1ß (IL-1ß, the primary endpoint), hemoglobin A1c (HbA1c), and high sensitivity C reactive protein (hsCRP), were performed at baseline and after 12 weeks.

**Results:**

Mean age of study patients was 67 ± 9 years, 75% were males, and baseline HbA1c and inflammatory markers levels were similar between the two groups. At 12 weeks of follow up, levels of IL-1ß, hsCRP, and HbA1c were significantly lower in the intervention group as compared with the control group. There was a continuous elevation of IL-1ß among the control group, which was not observed in the intervention group (49 vs. 4%, respectively; p < 0.001). The hsCRP was lowered by 60% in the vildagliptin/metformin group vs. 23% in the metformin group (p < 0.01). Moreover, a significant relative reduction of the HbA1c was seen in the intervention group (7% reduction, p < 0.03).

**Conclusion:**

The addition of vildagliptin to metformin treatment in patients with type 2 diabetes and CAD led to a significant suppression of the IL-1ß elevation during follow up. A significant relative reduction of hsCRP and HbA1c in the intervention group was also observed.

*Trial registration* NCT01604213

**Electronic supplementary material:**

The online version of this article (doi:10.1186/s12933-017-0551-5) contains supplementary material, which is available to authorized users.

## Background

Diabetes mellitus (DM) is one of the leading causes of death in the USA and Europe [1, 2] Patients with ischemic heart disease (IHD) and diabetes are at a particularly high risk for the recurrence of cardiovascular events. Cardiovascular disease (CVD) risk is two- to four-times greater in individuals with DM as compared to individuals without DM [2–4].

It is well known that DM induces complex vascular changes, promoting accelerated atherosclerosis and hypercoagulability, as can be assessed indirectly by a number of markers. Principal perturbations include endothelial dysfunction, increased inflammatory plaque infiltration, adhesion molecule over-expression and adverse effects of circulating fatty acids and advanced glycosylation end products [5, 6]. Consequently, diabetes is recognized as an independent risk factor for premature atherosclerosis, and for recurrent cardiovascular events in this population [7].

Much evidence supports a pivotal role for inflammation in all phases of atherosclerosis, from the initiation of the fatty streak to the culmination in acute coronary syndromes [8]. Inflammation also is involved in many of the metabolic abnormalities associated with diabetes, the most important of them being insulin resistance [3, 9].

IL-1 is the “apical” pro-inflammatory mediator in both acute and chronic inflammation [10]. It plays a major role in the activation of innate immunity [11], induces the synthesis and expression of multiple secondary inflammatory mediators including IL-6, IL-18 and IL-33 [12, 13], and is strongly associated with the development of atherosclerosis and impairment of cardiac function in diabetic patients [14].

Patients with diabetes have elevated levels of oxidized LDL (ox-LDL) in their macrophages, which further promotes the secretion of IL-1ß. Dipeptidyl peptidase-4 inhibitors (DPP4i, gliptins) were found to repress this elevation in animal studies [15–17].

High sensitivity C reactive protein, a well-known marker of inflammation, is produced by hepatocytes under regulatory control from circulating cytokines, in particular IL-1 and IL-6 [18].

Animal studies involving gliptins have suggested numerous beneficial anti-atherosclerotic effects, well beyond their primary role in lowering blood glucose [19, 20]. In addition, anti-remodeling effects have also been proposed [21], although this feature has not been established in a clinical setting. Concomitant treatment with a gliptins and metformin may offer an attractive glycemic reduction modality with a synergistic mechanism of action while exerting additional vascular protective benefits. The effect of gliptins on the above mentioned parameters has not been studied in humans.

Several pleiotropic beneficial effects of metformin beyond its glucose-lowering effect have been described previously [22, 23]. This compound improves the angiogenic functions of endothelial progenitor cells via various signaling pathways [24–27] and presents clear anti-inflammatory effects [27, 28], even irrespective of diabetes status [22, 29]. Accordingly, in the present study we designed a prospective randomized clinical trial in order to assess possible incremental anti-inflammatory and athero-thrombotic protective effects of combined vildagliptin–metformin therapy vs. metformin alone in a clinical setting. Specifically, we focused on the effects of DDPi therapy on IL-1ß due to its important role as a pro-inflammatory signaling cytokine, a key factor in the pathogenesis and progression of atherosclerosis [30–34].

## Methods

### Study design and patients

This was a 12-week, single-center, prospectively randomized, non-blinded, controlled study to provide evidence on the effects of vildagliptin on key biomarkers of athero-thrombosis and inflammation in a population of diabetic patients with coronary artery disease who undergo cardiac rehabilitation.

Participants eligible for this trial included males and non-child-bearing-potential females over the age of 21 who have (a) documented coronary artery disease >30 days; and (b) evidence of suboptimal type II diabetes control on the basis of Hemoglobin A1c (HbA1c) ≥6.5%, despite the use of oral anti-diabetic mono-therapy. Standard of care secondary prevention for coronary artery disease background therapy included, but was not limited to, lipid lowering, anti-hypertensive, ß blockers, and antiplatelet therapy, as appropriate and in accordance to current guidelines.

Patients were excluded if they had significant renal impairment (creatinine ≥1.4 mg\dL in female or ≥1.5 mg\dL in male patients), planned coronary intervention or planned surgical intervention (percutaneous coronary intervention or coronary artery bypass grafting), recent (<30 days) acute coronary syndrome (ACS), history of lactic acidosis, type I diabetes, current HbA1c >7.5%, or any significant hepatic, renal or cardiovascular medical conditions [35].

**Fig. 1:**
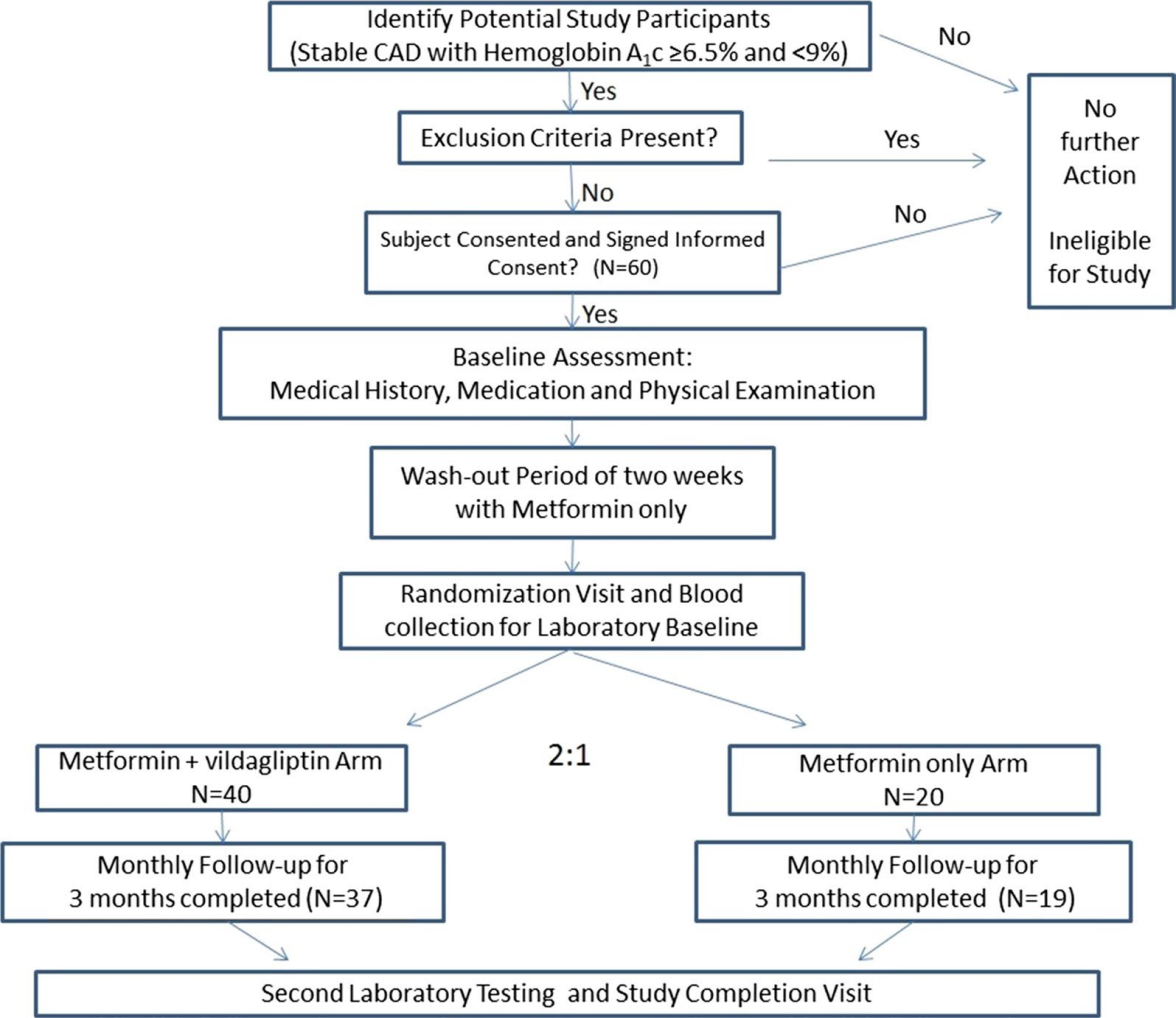
Study flow diagram

We prospectively enrolled 60 patients who met the study’s inclusion and exclusion criteria, and randomized them in a 2:1 ratio to either vildagliptin–metformin therapy (n = 40) or metformin therapy (n = 20). Study design and flow are presented in Fig. 1.

The study consisted of a 1–2 weeks screening period, followed by a 2-week wash-out period based on the current medication regimen.

Eligible patients (HbA1c ≥6.5 and ≤7.5%) who received current anti-diabetic mono-therapy (not including metformin or a gliptin), initially received substituted anti-diabetic treatment with metformin. Pre-specified substitution of oral anti-diabetic mono-therapy was permitted if clinically reasonable and safe. A washout period of 2 weeks took place prior to randomization. During this period, treatment with open label metformin was carried out with blood glucose monitored regularly.

Initial dose was 850 mg once daily, with a dose increase to a maximum of 850 mg TID with a target of fasting glucose ≤130 mg/dL. For patients who were eligible for the study who received current treatment with metformin mono-therapy, a dose increase was also allowed to a maximum of 850 mg TID aiming for a target of fasting glucose ≤130 mg/dL.

Eligible patients (HbA1c ≥6.5 and <9%) not on current anti-diabetic therapy initially received open-label metformin mono-therapy for a period of 2 weeks prior to randomization. During this period, treatment with metformin was carried out with blood glucose monitored regularly. Initial dose was 850 mg once daily, with a dose increase to a maximum of 850 mg TID to a target of fasting glucose <130 mg/dL.

### Study assessments and endpoints

The primary endpoint was change in the inflammation marker IL-1ß from baseline to week 12 or the final visit. Secondary efficacy assessments included weight reduction as well as changes in HbA1c, hsCRP, IL-1 alpha, IL-6, IL-10, tumor necrosis factor alpha (TNF-alpha), monocyte chemoattractant protein-1 (MCP-1)monocyte subsets by FACS, and matrix metallo-proteinase 9 (MMP-9). Safety assessments included recording and monitoring of treatment-emergent adverse events; biochemistry and hematology laboratory test results; and vital signs.

### Follow-up visits

All patients were invited for monthly follow-up visits with study coordinators. During these visits we monitored both clinical and adverse events, verified medication compliance and evaluated any hypoglycemic events. Changes in weight and in drug regimen were recorded. At the completion of the 3-month treatment, blood was drawn for laboratory testing as done at baseline. Blood samples did not contain identifying information and all tests were performed in a blinded fashion.

### Statistical methods and power calculation

The proposed sample size was calculated to demonstrate a significant improvement in the intervention group compared to the control group with at least 90% power and a two-sided 5% type 1 error.

Variables are expressed as mean ± standard deviation (SD) or median and inter quartile range (IQR). Categorical data are summarized as numbers and percentages. The demographic, clinical characteristics and laboratory values of patients at baseline according to the two pre-specified groups were compared with the use of the independent t test for normally distributed continuous variables, or non-parametric tests for covariates violating the normality assumption, and the Chi square test was used for comparison of categorical variables and for percentage changes.

In order to account for the possible effect of baseline parameters such as LDL levels and additional characteristics, we divided the continuous variable “percent change in IL-1ß” into three equal percentiles on scanned cases with roughly the same number of observations in each group. The lowest tertile represented the smallest increase. We used binary logistic regression modeling to assess the independent effect of vildagliptin (vs. metformin only) on the likelihood of IL-1ß changes beyond the lowest tertile (changes greater than recorded in the lowest tertile). The following covariates were introduced along with the vildagliptin vs. metformin only group: age, gender, serum creatinine, hypertension, heart failure, previous MI or past cerebrovascular accident.

Statistical significance was accepted for a two-sided p < 0.05. The statistical analysis was performed with IBM SPSS version 20.0 (Chicago, IL, USA) and SAS version 9.2 (SAS institute Inc.).

## Results

The disposition of patients from screening to study endpoint is depicted in Fig. 1. Of the 60 patients that were included in the study, 40 were randomized to the intervention metformin–vildagliptin group and 20 to the control metformin group. The percentage of randomized patients who discontinued the study was overall low yet somewhat higher in the vildagliptin group (5 vs. 9%; p = 0.13; respectively), mainly due to loss to follow-up (Fig. 1).

The demographic and baseline characteristics of the randomized patients were generally similar between the treatment groups (Table 1). The mean age was 67 ± 9 years, 75% male, and 61% had previous myocardial infarction. The only statistically significant difference between the two groups was that patients in the vildagliptin–metformin group had lower triglycerides levels compared to the metformin group (124 ± 41 vs. 176 ± 95; p < 0.001; respectively).

**Table 1:** Baseline characteristics of the study population by the two pre-specified groups

	Metformin group N = 20	Vildagliptin + metformin group N = 37	p value
Age (years)	68 ± 9	66 ± 9	0.85
Male gender	12 (60%)	31 (84%)	0.11
BMI	28 ± 4	28 ± 4	0.68
Past myocardial infarction	11 (55%)	22 (59%)	0.77
Status post CABG	7 (35%)	7 (20%)	0.28
Hypertension	16 (80%)	28 (77%)	0.77
TIA	2 (10%)	3 (8%)	0.58
Left ventricular EF	52 ± 10	53 ± 11	0.56
NYHA class ≥III	3 (15%)	9 (23%)	0.76
past valve repair/replacement	1 (5%)	1 (2.6%)	0.76
ACE inhibitors	12 (60%)	22 (59%)	0.94
ARBs	2 (10%)	10 (26%)	0.16
ß Blockers	17 (85%)	27 (74%)	0.35
Calcium channel blockers	5 (25%)	6 (16%)	0.37
Statins	19 (95%)	37 (100%)	0.63
Fasting glucose	139 ± 31	147 ± 50	0.71
AST	22 ± 7	28 ± 12	0.10
ALT	22 ± 8	29 ± 12	0.16
HDL	39 ± 7	41 ± 9	0.63
LDL	75 ± 20	76 ± 30	0.35
Triglycerides	176 ± 95	124 ± 41	0.001
HbA1c	7.3 ± 0.6	7.1 ± 0.5	0.63
IL-1 beta	37.5 ± 24	34.5 ± 11	0.58
IL-6	8.4 ± 5.5	6.7 ± 2	0.11
IL-10	8.2 ± 1	11.9 ± 3	0.22
TNF-alpha	15.6 ± 4.4	14.2 ± 2.5	0.12
MCP-1	180 ± 38	173 ± 43	0.58
Matrix metallo-proteinase 9	60,355 ± 2519	61,343 ± 17,181	0.82

*ACE* angiotensin converting enzyme, *ALT* alanine transaminase, *ARBs* angiotensin II receptor antagonists, *AST* aspartate transaminase, *BMI* body mass index, *CABG* coronary artery bypass grafting, *EF* ejection fraction, *HbA1c* hemoglobin A1c, *HDL* high density lipoprotein, *IL* interleukin, *LDL* low density lipoprotein, *NYHA* New York Heart Association, *MCP-1* monocyte chemoattractant protein-1, *TNF* tumor necrosis factor

Approximately three quarters of the patients in both groups were already treated with metformin prior to enrollment in the trial, with a mean dose of 1250 mg/day. The remaining 24% patients were started on metformin treatment for 3 weeks wash out period. All patients received 25 or 50 mg/daily dose of vildagliptin added to their regimen (based on their HbA1c).

### Efficacy and safety

#### Primary end point: IL-1ß

**Fig. 2:**
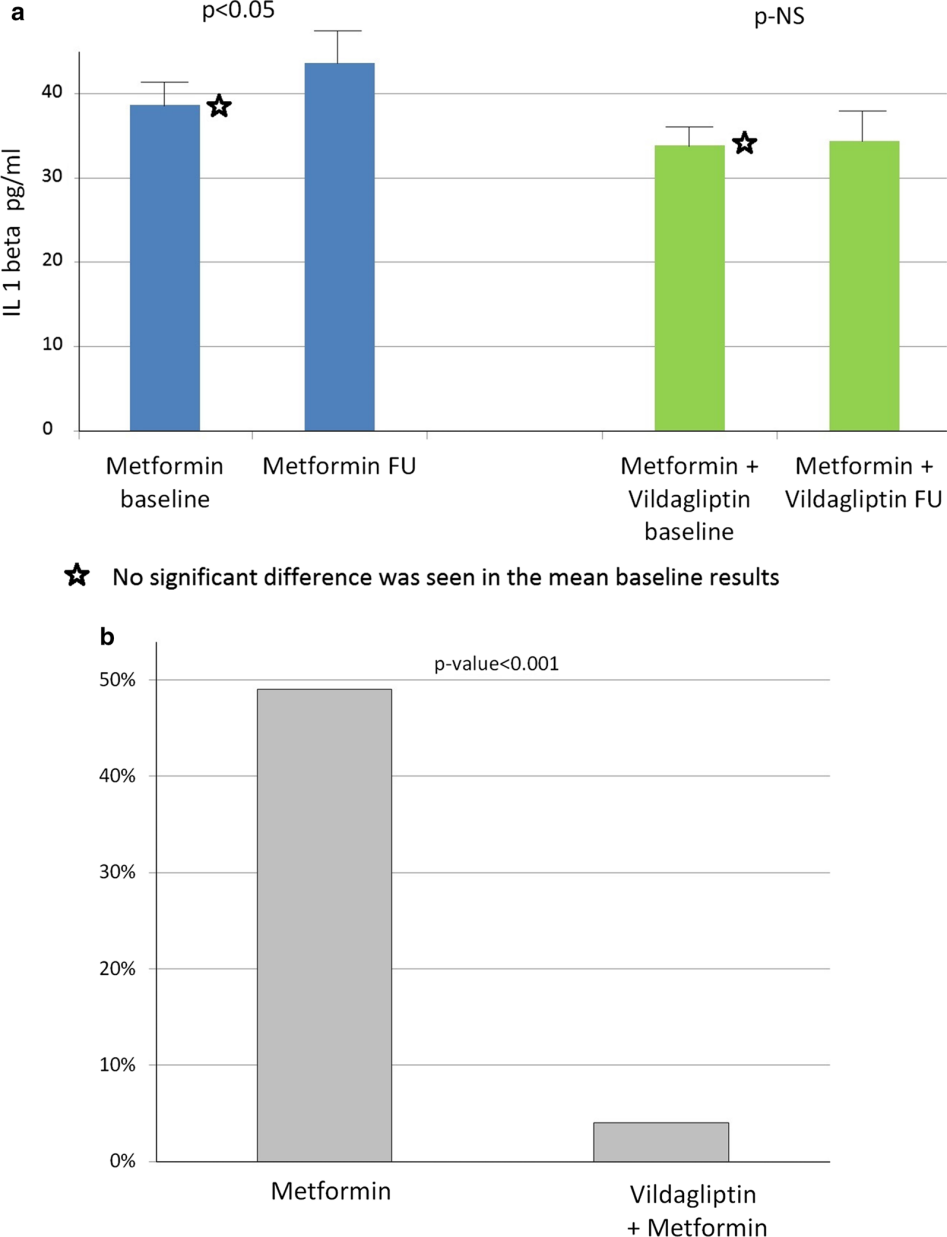
**a** Levels of IL-1ß at baseline and after 12 weeks of treatment; **b** Percent change of IL-1ß {Δ% = [(value after treatment−baseline value)/baseline value × 100]}

Figure 2a shows the distribution of the basal IL-1ß levels. There were no statistically significant differences in the basal values (mean of 35 pg/mL in the vildagliptin–metformin group vs. mean of 37 pg/mL in the metformin only group; p value = 0.58). Following 12 weeks of treatment, the levels IL-1ß were significantly greater in the metformin group than the combined group (44 vs. 34 pg/mL; p-value < 0.01, respectively; Fig. 2a).

Additionally, Fig. 2b shows the percent change in IL-1ß levels following the three months of treatment. During the 12 weeks of follow up, an increase of 49% was observed in the metformin only group compared to 4% change in the vildagliptin/metformin group (p < 0.001).

Consistently, multivariate binary logistic regression showed that vildagliptin treatment was independently associated with a 79% (p = 0.01) lower likelihood of an increase above the lower tertile of percent change in IL-1ß as compared to metformin-only therapy [OR 0.21 (95% CI 0.04–0.92);p = 0.01].

#### Secondary end points

**Fig. 3:**
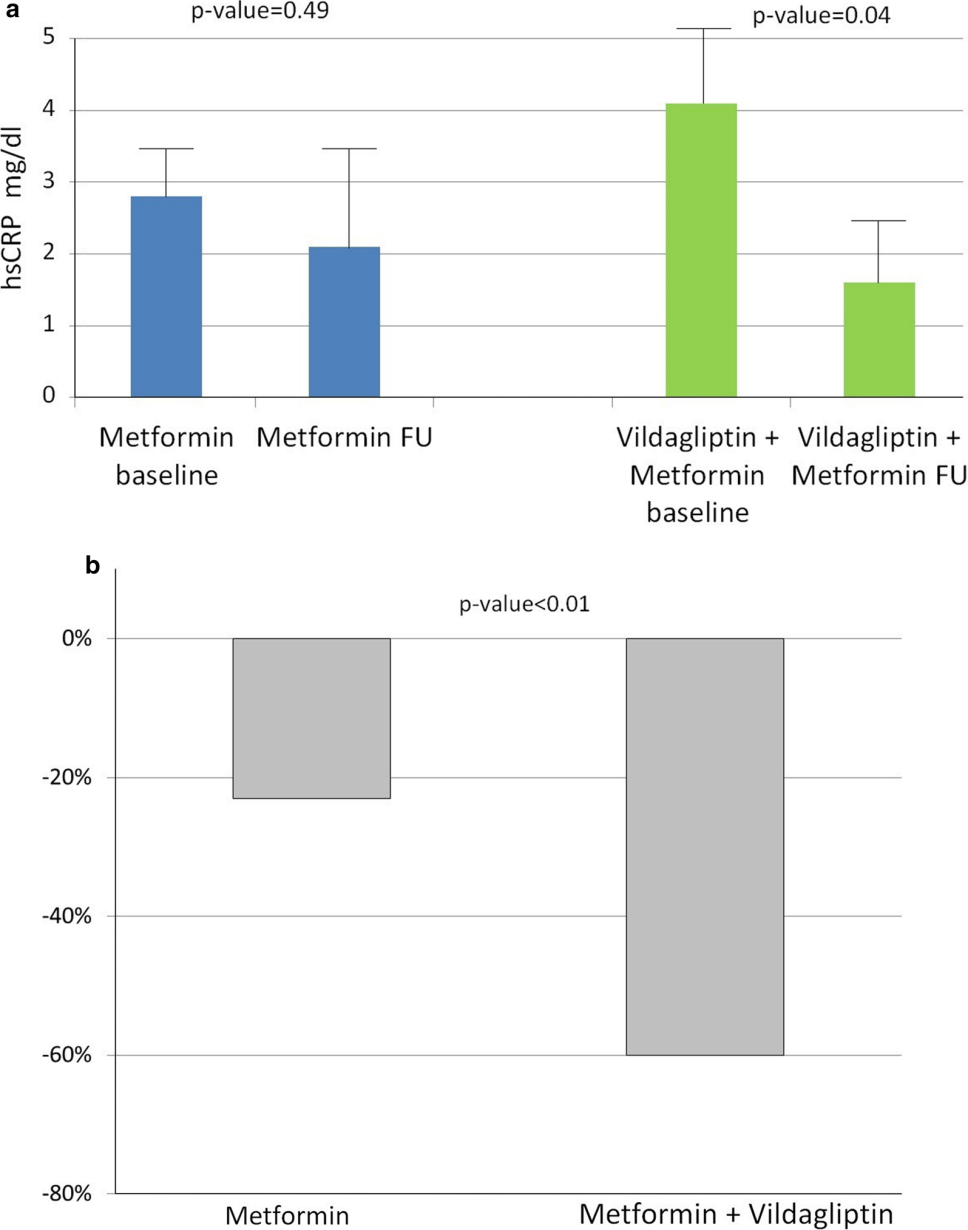
**a** hsCRP values at baseline and follow up **b** hsCRP percentage change after 12 weeks of treatment

A significant lowering of hsCRP levels was seen among the vildagliptin–metformin group. The hsCRP was lowered by 60% after the initiation of vildagliptin, as compared to only 23% lowering in the metformin group; p >  0.01 for the comparison (Fig. 3).

It is to mention, that three patients (two in the vildagliptin–metformin group and one in the metformin group were excluded, secondary to extreme high levels (more than 40 mg/dL, or more than 250% fold change) either at baseline or at follow up, indicating another concomitant disease such as infection, malignancy, etc.

**Fig. 4:**
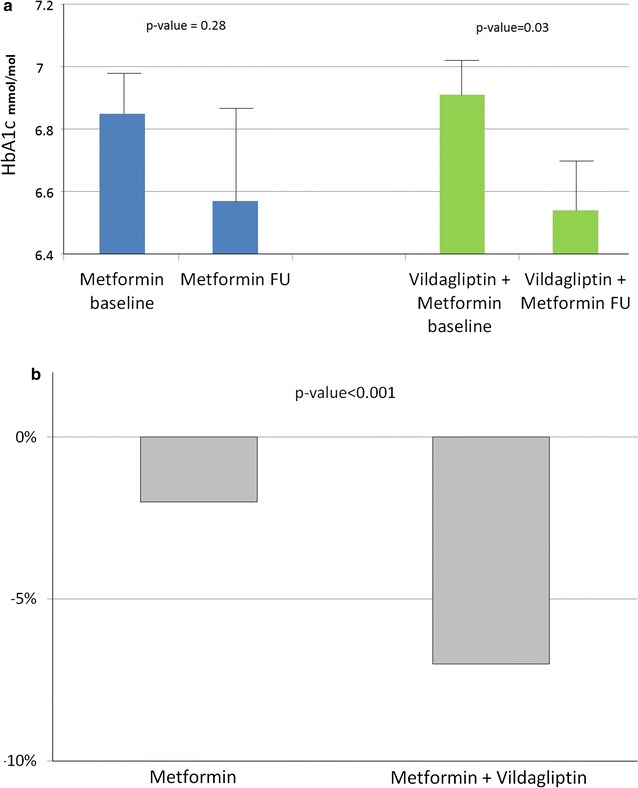
**a** HbA1c % values at baseline and follow-up **b** HbA1c percentage change after 12 weeks of treatment

The addition of vildagliptin resulted in a significant absolute reduction of HbA1c by 0.37% (7% percent change from baseline), compared with a smaller non-significant absolute reduction of 0.28% (2% percent change) in the metformin only group (Fig. 4).

Furthermore, a trend for lower results was also seen after the addition of vildagliptin in the other markers. Please see Additional file 1: Table S1, Additional file 2: Figure S1, Additional file 3: Figure S2.

#### Compliance and safety

The overall safety and tolerability of the addition of vildagliptin was very good, as no incidence of drug related adverse events were reported in both treatment groups, and no discontinuations were reported in both groups, except a 5-day discontinuation in the control intervention arm (case of gastroenteritis). Additionally, 1 patient had an episode of atrial fibrillation and 2 other patients visited the emergency department for atypical chest pain, and were discharged home after an acute coronary syndrome was ruled out. A total of 4 patients did not complete the study (1 in the control group and 3 in the intervention arm), 1 opted not to participate prior to randomization, 1 left the country, and the other 2 withdrew due to non-medical reasons after 1 month of treatment.

## Discussion

Our main finding in this study was that vildagliptin 50 mg bid added as an OAD to metformin 850–2550 mg prevented the elevation of IL-1ß during the study period, whereas a 49% elevation was observed in the metformin-only group.

Patients with diabetes have a continuous increase in IL-1ß since high concentrations of glucose stimulate IL-1ß production from the pancreatic ß cell itself, implicating a role for IL-1ß in type 2 diabetes. Moreover, the high levels of free fatty acids act together with glucose to stimulate IL-1ß production [36, 37]. In a randomized, placebo-controlled study of anakinra (IL-1 inhibitor), gene expression for IL-1ß was >100-fold higher in ß cells from patients with type 2 diabetes than from patients without. Subsequently, it was shown that patients who responded to Anakinra used 66% less insulin to obtain the same glycemic control. This observation suggests the functional restoration and partial regeneration of ß cells and the pivotal role of IL-1 [38]. Furthermore, interleukin-1 receptor antagonists were found to improve endothelial dysfunction in diabetic rats [39].

Additionally, diabetics have elevated levels of oxidized LDL (ox-LDL) in their macrophages, which further promotes the secretion of IL-1ß thus contributing to the positive feedback and new synthesis of IL-1ß. In several previous studies, DPP-4 inhibitors were found to repress this elevation; however, these were only animal studies [32–34] and to our knowledge we are the first to demonstrate these findings in stable diabetics patients with CAD.

Interleukin-1ß induces the synthesis and expression of numerous secondary inflammatory mediators and also induces its own production and processing, representing a key step in the pathogenesis of many auto-inflammatory diseases and the sustained increase of IL-1ß [10, 32].

Therefore, patients with both diabetes and atherosclerosis are especially prone to high levels and persistent increase in IL-1ß [15]. In our study, the addition of vildagliptin appears to inhibit this increase in IL-1ß and led to stabilization of the IL-1ß levels through the follow up period.

As already mentioned, there is a link between diabetes and atherogenesis which may be related to the high circulating levels of ox-LDL and AGEs, both of which induce endothelial dysfunction and thus inflammation [40]. Manica-Cattani et al. [17] have shown that macrophages treated with ox-LDL generate a number of cytokines, including IL-1ß. Liu et al. [16] have also shown that ox-LDL induces IL-1ß secretion promoting foam cell formation leading to atherosclerosis.

It is well-established that inflammation plays a major role in atherogenesis from a number of perspectives [41]. Inflammatory cytokines, including IL-1ß, induce a number of alterations in key steps leading to vascular injury, such as endothelial dysfunction, thrombosis and apoptosis. This inflammatory response is related mainly to the activation of the immune system, both the innate and the acquired, via these inflammatory cytokines [42].

Interleukin-1β (IL-1β), the main active form of the IL-1, is a prototypic multifunctional cytokine which plays a significant role in promoting inflammation, with a subsequent important effect on the pathogenesis and progression of atherosclerosis [43].

Recent studies in the field have shown that DPP-4 inhibitors block the catabolism of GLP-1R agonists, leading to toll-like receptor 4 (TLR4) activation and decreasing PKC activity [15, 34, 44]. Although the mechanism is still not known, DPP-4 inhibitors repress the expression of TLR4 and lead to decreased activation of PKC, leading to the suppression of the overproduction of IL-1ß found in ox-LDL treated human macrophages [15, 16].

Our study supports the observations of Yao-Dai et al. [15] showing a repression of IL-1ß via GLP-1 receptor inactivation in macrophages exposed to DPP-4 inhibitors. However, it should be noted that these prior studies were performed in vitro, with direct injection of DPP-4 into the macrophages. To the best of our knowledge, this is the first clinical study to demonstrate these findings in patients with therapeutic doses of the DPP-4 inhibitor vildagliptin. Moreover, the patients in our study were well treated with statins, ß-blockers, metformin, and were actively undergoing cardiac rehabilitation and nutritional consultations. Despite all these protective effects, the addition of vildagliptin significantly reduced the expression of IL-1ß in these patients making our results even more robust.

Among all other inflammatory markers (except hsCRP), there was an increase from baseline levels through the follow up period. The use of DPP4 was associated with a trend for better suppression of these elevations, despite almost normal levels at baseline. We believe the effect of vildagliptin did not reach significance because of the cohort size and relatively short follow-up period.

Although it failed to achieve significance, the change in hsCRP levels among the vildagliptin group was at least double as much as the change in the metformin-only group (50% decrease vs. 20% decrease; p = 0.13; respectively). We believe there is a strong correlation, but we failed to reach significance because of the above mentioned limitations of the study.

Moreover, our patients are well treated stable patients, who participate regularly in our cardiac rehabilitation institute, with monitored follow up, and frequent physician, dietitian and physiologist interactions. Furthermore, they received potent statins and additional secondary prevention measures according to the latest national guidelines.

## Conclusion

Compared to metformin only, the addition of vildagliptin led to a significant suppression of the IL-1ß elevation in patients with established CAD receiving an optimal secondary prevention regimen.

A significant relative reduction of hsCRP and HbA1c in the intervention group also was observed.

As IL-1ß is a key regulator in the inflammation and atherosclerotic process, this effect could be possibly associated with improved clinical outcomes. Larger studies with longer follow-up periods are necessary to further explore these findings.

## Additional files

**Additional file 1: Table S1.** Showing the percentage change {Δ% = [(value after treatment - baseline value)/baseline value * 100]} in each group of patients after 12 weeks of treatment.

**Additional file 2: Figure S1.** No difference in %CD14 was found between treatment and control groups.

**Additional file 3: Figure S2.** No significant difference in %CD16 was found between treatment and control groups.

## Abbreviations

ACS: acute coronary syndrome
CAD: coronary artery disease
CVD: cardiovascular disease
HbA1c: hemoglobin A1 C
hsCRP: high sensitive C reactive protein
DM: diabetes mellitus
DPP4i: dipeptidyl peptidase-4 inhibitors
IL-1ß: interleukin 1 beta
IHD: ischemic heart disease
ox-LDL: oxidized low density lipoprotein
TLR4: toll-like receptor 4
