# Supplementary material for: The addition of vildagliptin to metformin prevents the elevation of interleukin 1ß in patients with type 2 diabetes and coronary artery disease: a prospective, randomized, open-label study
Source: Cardiovasc Diabetol. 2017 May 22;16:69. doi: 10.1186/s12933-017-0551-5 (PMC5440983; doi:10.1186/s12933-017-0551-5)
Supplement: Supplementary file 1 — Additional file 1: Table S1. Showing the percentage change {Δ% = [(value after treatment - baseline value)/baseline value * 100]} in each group of patients after 12 weeks of treatment. [file 12933_2017_551_MOESM1_ESM.docx]

**Table S1 –** showing the percentage change {Δ% = [(value after treatment - baseline value)/baseline value * 100]} in each group of patients after 12 weeks of treatment.

|  | **Metformin Group N=20** | **Vildagliptin+Metformin Group N=37** | **p=value** |
| --- | --- | --- | --- |
| IL – 6 | 120% ± 60% | 95% ± 20% | 0.18 |
| IL – 10 | 104% ± 16% | 94% ± 23% | 0.14 |
| TNF – alpha | 96% ± 21% | 95% ± 13% | 0.83 |
| MCP-1 | 101% ± 17% | 96% ± 15% | 0.29 |
| Matrix metallo-proteinase 9 | 97% ± 7% | 98% ± 7% | 0.65 |

IL = Interleukin; MCP-1 = Monocyte Chemoattractant Preotein-1; TNF = Tumor Necrosis Factor.
