# Supplementary figures and images for: The addition of vildagliptin to metformin prevents the elevation of interleukin 1ß in patients with type 2 diabetes and coronary artery disease: a prospective, randomized, open-label study
Source: Cardiovasc Diabetol. 2017 May 22;16:69. doi: 10.1186/s12933-017-0551-5 (PMC5440983; doi:10.1186/s12933-017-0551-5)

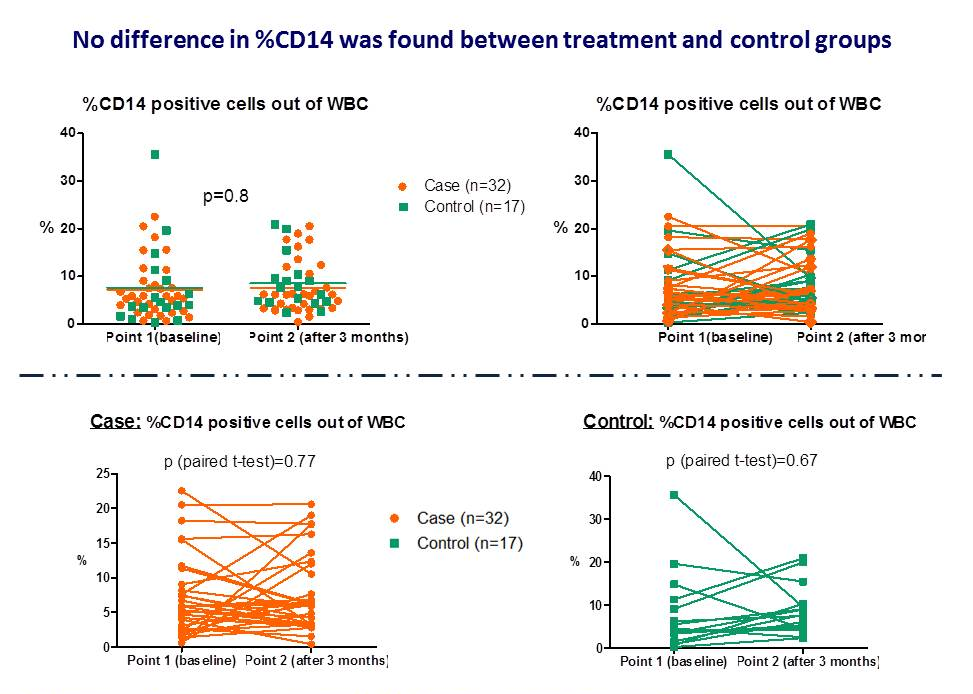

Supplement: Supplementary file 2 — Additional file 2: Figure S1. No difference in %CD14 was found between treatment and control groups. [file 12933_2017_551_MOESM2_ESM.tif]

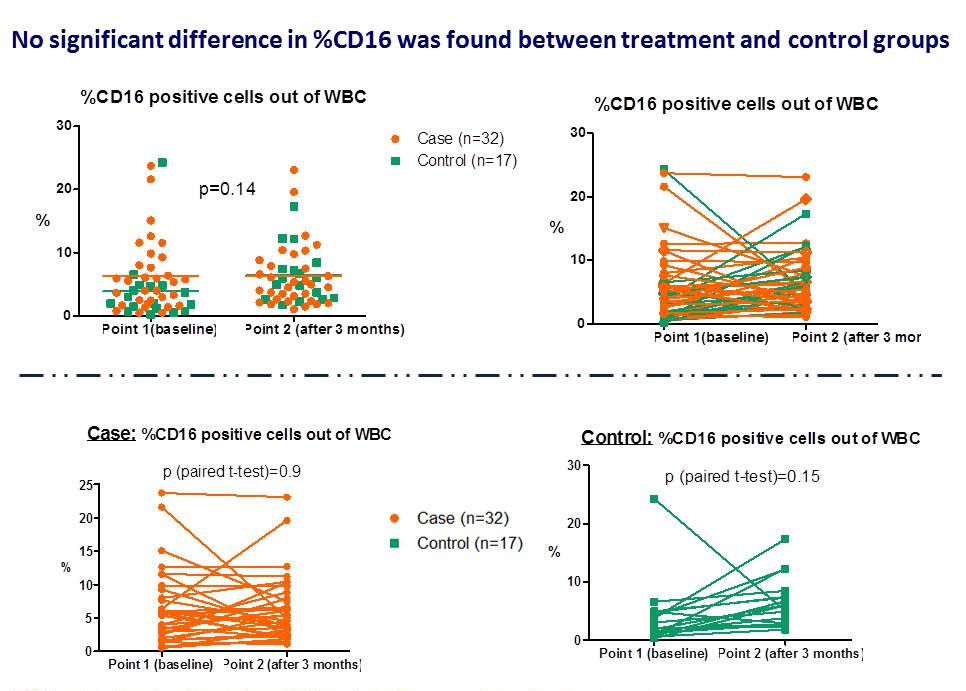

Supplement: Supplementary file 3 — Additional file 3: Figure S2. No significant difference in %CD16 was found between treatment and control groups. [file 12933_2017_551_MOESM3_ESM.tif]
